# An Unusual Trigger of Grover’s Disease (GD)

**DOI:** 10.7759/cureus.40648

**Published:** 2023-06-19

**Authors:** Shaniza Haniff, Maria E Butler, Elias A Abou-Jaoude, Mary L Lenahan

**Affiliations:** 1 Internal Medicine, The University at Buffalo Internal Medicine Training Program at Sisters Hospital, Buffalo, USA; 2 Allergy and Immunology; Dermatology; Pathology, Lake Erie College of Osteopathic Medicine, East Amherst, USA; 3 Internal Medicine/Resident Physician, University at Buffalo, Buffalo, USA; 4 Dermatology, Sisters of Charity Hospital, Buffalo, USA

**Keywords:** honeybee sting, immune-mediated dermatosis, etiopathogenesis, allergens, transient acantholytic dermatosis, type 2 inflammation, grover’s disease

## Abstract

Grover’s disease (GD) is a rare skin condition that presents as a pruritic, erythematous papular, or papulovesicular rash. We report a unique case of GD triggered by honeybee stings. An 80-year-old Caucasian male presented with a pruritic papulovesicular rash on his trunk and arms after being stung by honeybees. He had a history of honeybee venom allergy and developed immediate erythema at the sting sites, which progressed over two days. His laboratory tests were unremarkable, including a complete blood count and comprehensive metabolic profile. Despite using oral antihistamines, emollients, and topical steroids, his rash continued to progress onto his neck, face, scalp, and back. A skin biopsy of the rash revealed suprabasilar and intraspinous acantholysis with focal corps ronds and upper dermis lymphocytic infiltrate -- the histopathologic finding of GD. He had failed first-line treatment for GD. However, after five months and significant morbidity, he was successfully treated with systemic steroids, high-potency topical steroids, emollients, and antihistamines for extensive and prolonged GD. This case report highlights honeybee venom as a possible trigger of GD and discusses a potential immune-mediated etiopathogenesis, which can be used to guide further research and management of this rare disease.

## Introduction

Grover’s disease (transient and persistent acantholytic dermatosis) is a rare skin condition that has not yet been fully described. It was first mentioned in the medical literature in 1970 by Ralph Grover. It was initially described as a transient condition, but subsequent reports have shown that it can last several months or be recurrent [1]. This has led to the name transient and persistent acantholytic dermatosis, more commonly known as Grover’s disease (GD). The rash of GD most commonly occurs on the trunk, and if inclusive of an additional body part, it is called extensive GD. The rash is often intensely pruritic, appearing as erythematous papules, keratotic papules, papulovesicular, or papulosquamous plaques that can erode or crust [1-2].

Grover’s disease most commonly affects middle-aged to elderly white men, with men being 3:1 times more likely to be affected than women [2]. It occurs in about 0.1% of the population [3]. Given the rarity of the disease, the etiology and pathogenesis are not fully understood [4]. However, documented triggers have been described and include heat, sweating, ionizing irradiation, end-stage kidney disease, prolonged bed rest, and solid organ transplantation [1]. The diagnosis of GD can be made clinically and supported with a skin biopsy of the rash showing acantholysis (the dissociation of keratinocytes from one another in the epidermis), a key histopathologic feature of the disease [5]. Clinically, GD may resemble other immune-mediated dermatological conditions, raising the question of immune-mediated etiopathogenesis [6-7]. We report a case of GD that highlights honeybees as an unusual trigger and describes the potential role of immune-mediated etiopathogenesis. 

## Case presentation

An 80-year-old Caucasian male presented with a pruritic rash after being stung by honeybees. He was stung on his bilateral arms and the right side of his chest, resulting in immediate edema and erythema around the sting sites. He had no mucosa involvement, including wheezing, swelling of the face or throat, or systemic symptoms like fever, chills, malaise, or lymph node swelling. The immediate skin reaction at the bee-sting site prompted him to use an epinephrine pen prescribed to him for a bee-venom allergy. Over the next two days, a pruritic, erythematous rash appeared on the bilateral arms and trunk, extending beyond the sting sites, leading him to see his primary care physician (PCP).

Four years prior, he had a single sting by a honeybee, resulting in an extensive local reaction (circular swelling greater than 10 cm) around the sting site accompanied by hives and itching on the trunk and extremities. His symptoms resolved within one day after using an epinephrine pen prescribed by his PCP. He was since diagnosed with a honeybee venom allergy. 

His medical history includes Hashimoto’s thyroiditis and degenerative joint disease. He takes levothyroxine and occasionally uses non-steroidal anti-inflammatory drugs, last used six months prior to this event. He denied cough, sore throat, chills, diarrhea, fevers, joint aches, and any contact with a similar rash. He also denied starting new medication or personal care products.

His laboratory work was normal, including a complete blood count, comprehensive metabolic panel, and thyroid function tests. His PCP prescribed an oral antihistamine (diphenhydramine) and an emollient cream for his rash. Despite treatment, the rash progressed to his neck and face over one month and was associated with severe pruritis. This prompted the patient to see a dermatologist.

**Figure 1:**
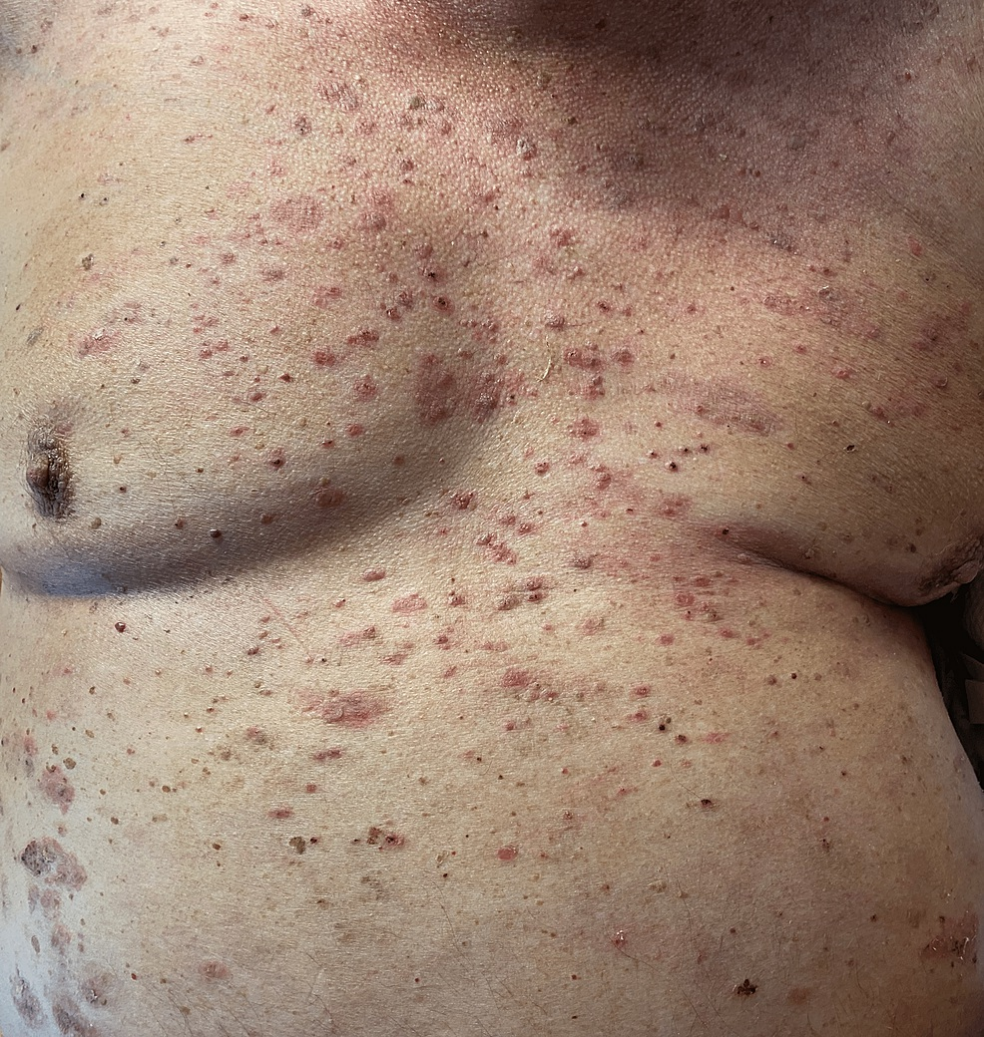
Well-defined erythematous papulovesicular eruption on the trunk.

A physical exam at the dermatologist visit revealed a pruritic, wide-spread, well-defined erythematous papulovesicular rash distributed on bilateral arms, trunk, neck, face, ears, and scalp (Figure 1).

**Figure 2:**
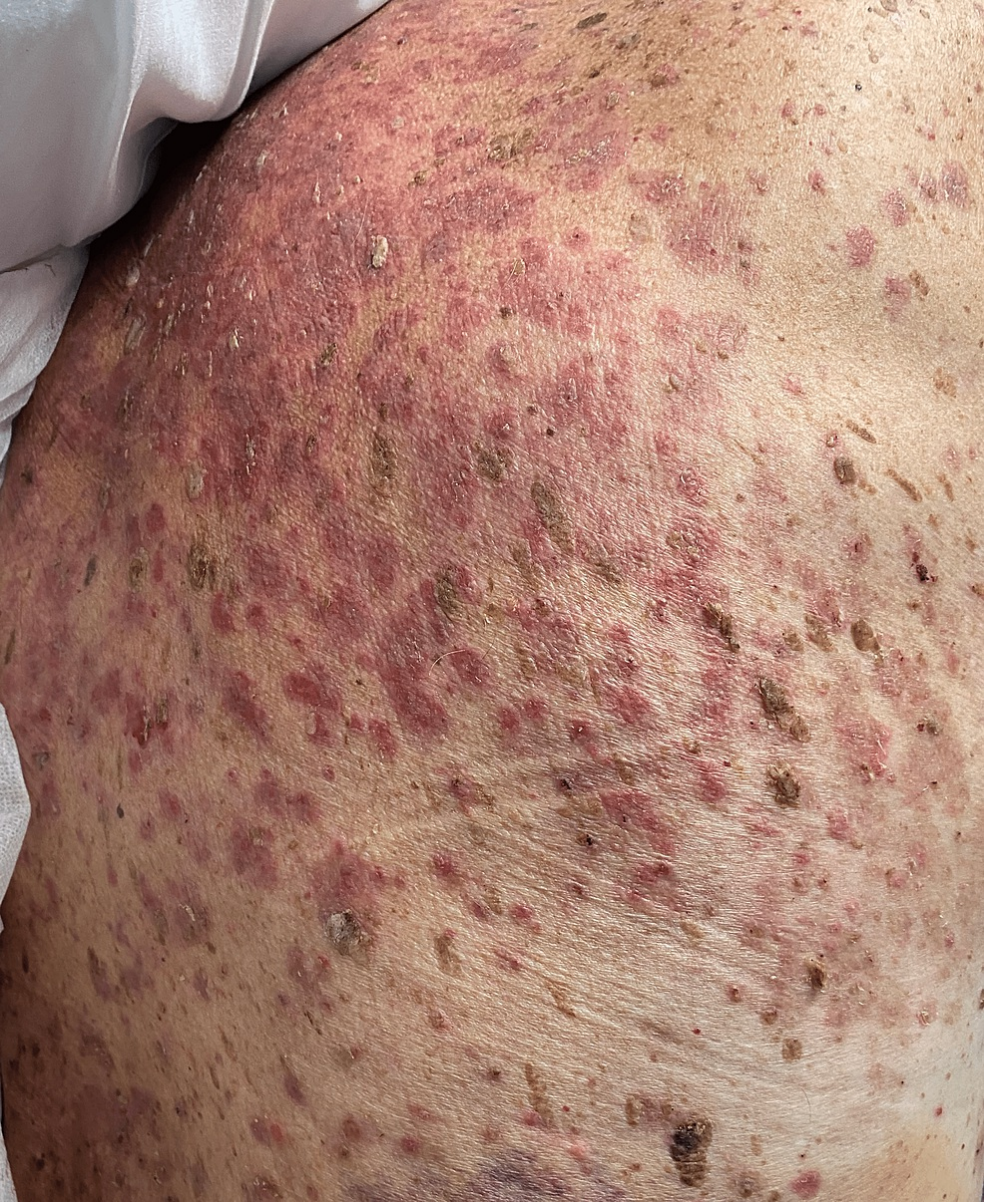
Well-defined erythematous patches and plaques on the back.

The rash had associated crusting, likely due to excoriation. The dermatologist prescribed a medium-strength steroid cream (triamcinolone acetonide 0.1%) for a presumed interface dermatitis reaction to bee stings. At the follow-up appointment three weeks later, the rash had progressed to well-defined erythematous patches and plaques on the back and new areas of scabbing on his trunk due to severe itching (Figure 2).

The patient described constant itching unless he used the prescribed topical cream. He has subsequently prescribed a lotion (pramoxine 1%) for pruritic relief and a high-potency steroid cream (clobetasol propionate 0.05%). A 4-mm skin punch biopsy of the left superior-lateral lower back was taken at this visit.

**Figure 3:**
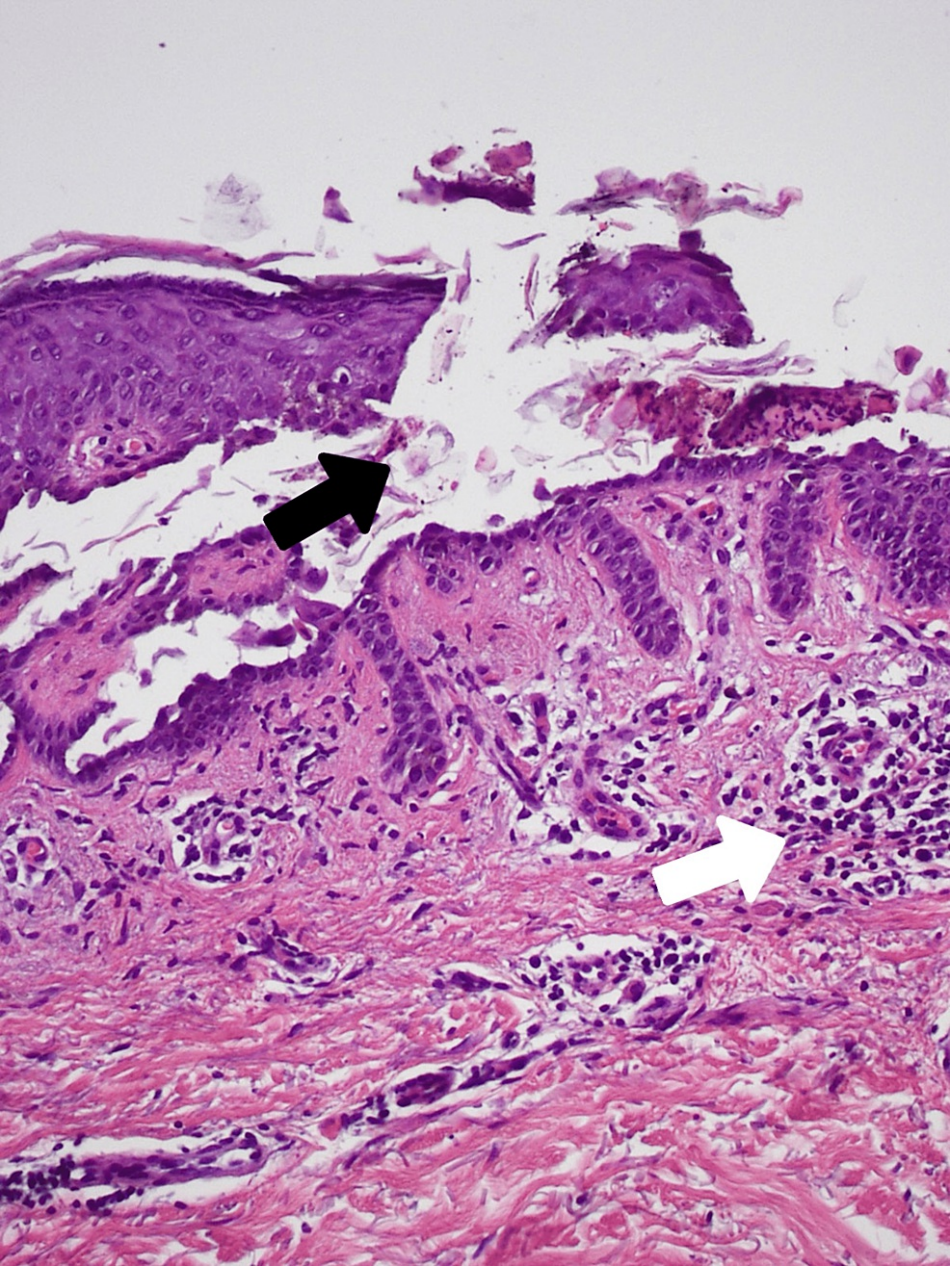
Hematoxylin and eosin (H&E) staining of suprabasilar acantholysis evidenced by the loss of coherence of keratinocytes (black arrow) and chronic inflammation evidenced by mononuclear inflammatory infiltrates in the dermis (white arrow). Magnification 20x.

**Figure 4:**
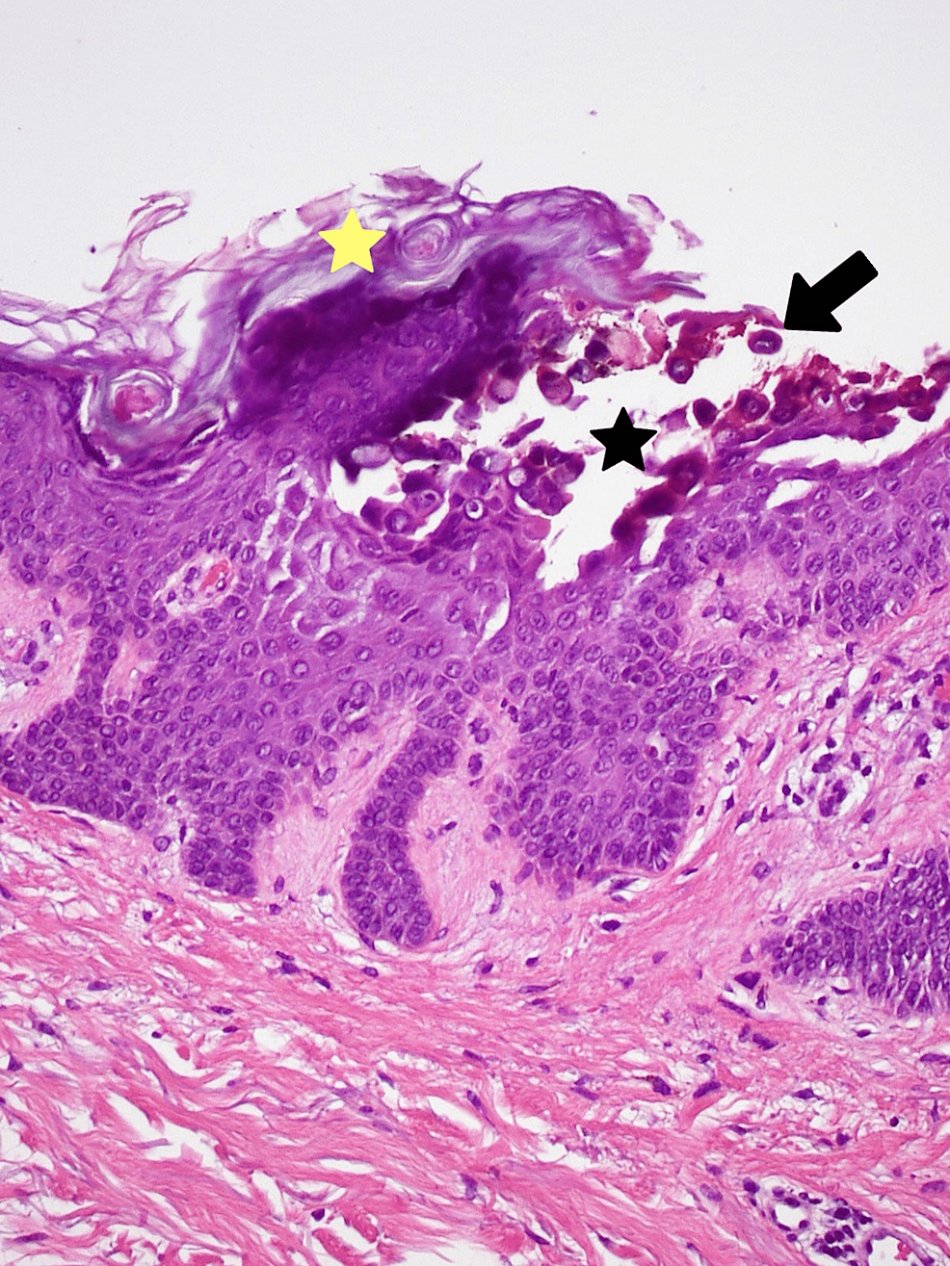
Hematoxylin and eosin (H&E staining) of hyperkeratosis (yellow star), intraspinous acantholysis (black star), and corps ronds (black arrow). Magnification 20x.

His skin biopsy revealed hyperkeratosis, suprabasilar and intraspinous acantholysis with focal corps ronds as well as upper dermis lymphocytic infiltrate consistent with the histopathologic findings of GD (Figures 3-4). At this point his rash was distributed on the trunk, arms, neck, face, ears, scalp, and back, covering 75% of his body surface. He was given an oral antihistamine (cetirizine) and systemic steroids (prednisone) for his confirmed diagnosis of GD. The patient’s extensive and prolonged course of GD rash improved over five months of this treatment. 

## Discussion

This report describes an extensive and prolonged case of GD that presented following multiple honeybee stings. GD is currently classified as a non-immune acantholytic dermatosis [7]. However, studies have proposed an immune mechanism for its etiopathogenesis [7]. Honeybee venom contains allergenic proteins that can trigger the immune system, resulting in a hypersensitivity reaction with dermatological manifestations [8].

The first exposure to the honeybee sting sensitizes the immune system by priming the venom allergen-specific immunoglobulin E (IgE) on high-affinity FcRI receptors of inflammatory cells [8-9]. This sensitization process may have occurred when our patient was stung four years prior with honeybee-specific venom. On subsequent exposures, the allergen cross-links the receptors and releases mediators, including histamine, causing immediate local edema and erythema of the skin [9]. Interestingly, the venom-specific allergen activates a strong type 2 inflammation, increasing interleukins and cytokines to act on epidermal (skin) cells. In individuals with a known allergy, such as in our case, the hypersensitivity reaction can be biphasic. The first results in an immediate skin reaction, and the second occurs within 72 h, evident in our patient two days later when he developed a pruritic, erythematous rash [8].

In the elderly population, the immune system changes to a predominantly pro-allergenic type 2 inflammation due to increased inflammatory mediators and antigen-presenting cell dysfunction [10]. These age-related changes to the immune system can give insight into why the incidence of GD increases with age [10]. The clinical presentation and distribution of the rash in GD can mimic other immune-mediated dermatological conditions, including pemphigus vulgaris, Hailey-Hailey disease, and Darier’s disease. However, the characteristic histopathological pattern of acantholysis (loss of cohesion between keratinocytes within the epidermis) on skin biopsy, along with the clinical presentation, is key in diagnosing GD [1].

Due to the rarity of GD, there have been no published randomized clinical trials of treatment. Nearly all management of GD comes from case series, case reports, and clinical experience [1]. First-line treatment includes topical steroids, vitamin D analogs, and oral antihistamines. In prolonged or extensive GD cases, systemic corticosteroids, retinoids, and phototherapy may be used [11]. Our patient successfully responded to systemic steroids, high-potency topical steroids, emollients, and antihistamines after a prolonged and extensive course. Prednisone suppresses the immune system by altering gene expression to decrease inflammatory cytokines and decreasing the migration of inflammatory polymorphonuclear lymphocytes [12]. Cetirizine, a selective antihistamine, also helps to calm the overactive immune response. If GD results from the overactivation of type 2 inflammation, the anti-inflammatory mechanism of both prednisone and cetirizine may explain why these drugs helped resolve this prolonged course of the disease.

In contrast to our study, other cases have reported extensive GD refractory to these treatment modalities, including systemic steroids. Drugs targeting the immune system have shown promise in treating GD. Dapsone and Dupilumab both inhibit type 2 inflammation and were successfully used in cases of severe GD management [2, 13]. Another drug, Etanercept, frequently used in autoimmune conditions, works by blocking cytokines in type 2 inflammatory pathways and has been effective in refractory GD in one case report [14]. Therefore, exploring an immune-etiopathogenesis of GD, particularly due to type 2 inflammation, can pave the way to improve treatment options and lead to a speedier recovery.

## Conclusions

This case report describes an extensive and prolonged course of GD in an elderly male patient. It highlights a potential new allergenic trigger, honeybee stings, and offers insight into immune-mediated pathogenesis. The role of type 2 inflammation in the etiopathogenesis of GD still requires further research and may provide clinical guidance in the early and successful management of GD. 
